# Hydrocolloid food additive intake in the Norwegian population: a dietary exposure assessment

**DOI:** 10.1007/s00394-026-04159-5

**Published:** 2026-11-11

**Authors:** Åsne Skram Trømborg, Jon Olav Vik, Lene Frost Andersen, Monica Hauger Carlsen, Harald Carlsen

**Affiliations:** 1 Faculty of Chemistry, Biotechnology and Food Science, Norwegian University of Life Sciences (NMBU), Ås, Norway; 2 Department of Nutrition, Institute of Basic Medical Sciences, Faculty of Medicine, University of Oslo (UiO), Oslo, Norway

**Keywords:** Food additives, Hydrocolloids, Exposure estimates, Habitual intake

## Abstract

**Purpose:**

Hydrocolloid food additives are abundant in foodstuffs and found across many food groups. Recent studies suggest that certain hydrocolloids may exert negative health effects, but quantitative data regarding their intake in the population remains scarce. The purpose of this study was to map the dietary intake and exposure prevalence of 15 hydrocolloid food additives in the Norwegian population, providing a foundation for research linking dietary intake to health outcomes.

**Method:**

Data were obtained from Norkost 4, a nationwide, cross-sectional dietary survey utilizing two non-consecutive 24-hour dietary recalls among 1964 Norwegian adults. The presence of 15 hydrocolloid additives in the food items consumed was identified through ingredient lists, and concentration data were derived from the European Food Safety Authority (EFSA).

**Results:**

Dietary exposure to hydrocolloids was widespread in the cohort but varied across individual hydrocolloids and food categories. Guar gum demonstrated the highest exposure prevalence (92%), whereas others, such as methyl cellulose, were rarely encountered. Demographically, the characteristics of high-tier consumers were highly additive-specific, indicating that no single, homogeneous profile of an “extreme additive consumer” exists. Furthermore, comparisons with international cohorts underscore how the choice of analytical framework and concentration data impacts estimation of exposure outcomes, making cross-country comparisons challenging.

**Conclusion:**

This comprehensive assessment provides the first mapping of hydrocolloid intake, and the first individual-level exposure estimation of food additives in general, within the Norwegian population. Ultimately, as long as brand-specific, public concentration data remains restricted, the precise quantification of real-world hydrocolloid exposure will remain a profound methodological challenge.

**Supplementary Information:**

The online version contains supplementary material available at https://doi.org/10.1007/s00394-026-04159-5.

## Introduction

Over 300 food additives are approved for use in the EU through Regulation (EC) No 1333/2008 [1]. These substances are added to food for specific technical purposes, as outlined in Annex I of the regulation. For example, “sweeteners” are defined as “substances used to impart a sweet taste to foods or in table-top sweeteners,” and “thickeners” are defined as “substances which increase the viscosity of a foodstuff.” The range of additives has proliferated alongside products with new and varying technical properties, with each additive offering distinct properties suited to specific applications such as thickening, gelling, emulsification, and stabilization. Although these substances are added to food for technological purposes, their chemical properties also determine how they behave upon ingestion, and in particular, how they interact with the gastrointestinal tract. Some of these substances are long polysaccharides derived from different sources, such as algae, plants, and bacteria [2]. These are used as thickening agents, emulsifiers, gelling agents, stabilizers, and fat replacers, resist degradation in the small intestine, and thus reach the colon undigested. In the colon they may serve as food for colonic bacteria and potentially alter the gut microbiota composition, function, and possibly affect the physicochemical properties in the intestinal lumen. They are often referred to as hydrocolloids. Examples include the additives carrageenan, xanthan gum, guar gum, carboxymethylcellulose (CMC), and pectin.

Even though the approval of food additives is based on thorough safety assessments and are regularly re-evaluated [3–14], several of the food hydrocolloids have gained attention due to studies suggesting a negative health impact. For example, recent epidemiological observational studies link intake of celluloses to cardiovascular disease [15] and carrageenan, guar gum, gum arabic, and xanthan gum to type II diabetes [16]. Experimental studies support some of these findings. In mice, carrageenan and CMC may worsen colitis, alter gut microbiota composition, and exacerbate metabolic syndrome [17–21]. In humans, carrageenan has been shown to increase intestinal permeability and reduce insulin sensitivity and trigger early relapses in ulcerative colitis patients [22–24]. Conversely, some hydrocolloids, for instance, gellan gum and guar gum, have demonstrated potential in ameliorating fatty liver disease [25] and colitis symptoms in mice [26], respectively.

However, the reported effects are in general small and subtle and should be interpreted with caution regarding clinical relevance. Further, The Norwegian Scientific Committee for Food and Environment in Norway (VKM) concluded in their systematic review on gastrointestinal effects of several of these hydrocolloids that existing studies are too few and the evidence is too weak for risk assessment of effects on the gastrointestinal tract in humans [27, 28].

While traditional risk assessments ensure basic safety, they are generally not designed to capture mild, but yet meaningful effects, such as how chronic exposure might interact with the gut microbiota and indirectly affect the host. This nuance is increasingly important given the widespread presence of hydrocolloids in foods [29, 30], where ultra-processed foods (UPF), rich in such additives, can account for up to over 50% of total energy intake [31–35]. Consequently, there is a growing interest in understanding the broader impact of these additives. However, actual population-level intake remains virtually unknown, as standard dietary assessments rarely quantify individual hydrocolloids.

Of the hydrocolloids, only carrageenan has been assigned Acceptable Daily Intakes (ADIs). ADIs are estimates of how much can be consumed daily over a lifetime without posing a significant risk to human health [36]. Whether or not such estimates should be based on toxicological assays or require other methodology, there is a pressing need for robust intake data to evaluate whether actual consumption is consistent with estimates from regulatory authorities (e.g. EFSA), and to identify potentially vulnerable population groups with elevated intake levels. Also, there is a growing interest in understanding the broader biological impact of these additives. However, systematic exposure data remain scarce and are currently limited to selected cohorts in France [37] and the US [38]. To date, no such data exists for Nordic populations, where dietary habits and food formulations may differ.

The present study aimed to identify and quantify the intake of 15 hydrocolloid food additives in the Norwegian population by combining product-specific additive-presence-data with Norkost 4 dietary recall data and EFSA concentrations. The 15 chosen hydrocolloids are the most prevalent on the Norwegian food market [30]. We further characterized food group contributors to exposure, examined variation in intake frequency and amount across demographic subgroups, including gender, age, BMI, and education level. We also identified co-consumption of additives and estimated habitual intake distributions for the most often consumed additives.

## Methods

### Diet survey

The present study used data from the recent national Norwegian diet survey among adults, Norkost 4. Norkost 4 was assessed as not subject to submission requirements by the Regional Committees for Medical and Health Research Ethics (REK; application number 469437) and was subsequently reported to the Service Provider for the Knowledge Sector (SIKT, reference number 265058). Extraction from the National Population Register was approved by the Norwegian Tax Administration (reference number 2022/5665038), and the Contact and Reservation Register authorized the retrieval of mobile phone numbers for the selected sample. The project was also approved by the Data Protection Officer at the University of Oslo.

The inclusion criteria in Norkost 4 were age between 18 and 80 years, holding a Norwegian ID number, and residence in Norway. Individuals living in institutions were not included. Dietary intake was assessed using two telephone-administered 24-hour recalls, distributed across both weekdays and weekends. A total of 4863 individuals were randomly selected and invited to participate in the survey, of whom 1964 completed both dietary recalls. Detailed information about the methodology is available in the main report [39] and characteristics about the study population are presented in the results section.

### Exposure estimation

Hydrocolloid intake was estimated by linking individual food consumption data from Norkost 4 to a product-level database of Norwegian food items, identifying the presence of hydrocolloids, assigning additive-specific concentrations, and calculating individual-level intake. Details in each step are described below.

#### Food additive identification

A database of specific food products available on the Norwegian market was generated in our previous study [30]. Briefly, food product information was retrieved from two food information databases, *Vetduat.no* and *Kassalapp*^®^, on October 21, 2024. Ingredient lists were screened for the additives using E-numbers, plain-text names, and common synonyms, accounting for frequent misspellings through regular expression searches. Each additive was counted once per product, even if listed multiple times across components in composite products.

The food items registered by the participants in Norkost 4 were matched with products available on the market, including information about their corresponding food additives. For many of the registered food items in Norkost 4, only a single corresponding product was found, while for others, there was a wide range of potential matches. In the latter case, all matching products were included, and the total registered intake was equally divided between them. This is illustrated in Table 1, where the generic entry “Beetroot salad”, registered without a specific product name, was matched to three different products on the market, each with partly different hydrocolloid content. Because no product name was specified by the participant, the total intake of 15 g was distributed equally across the three matching products (5 g each). In cases where a participant specified a product name, the entire registered intake was allocated to that single product.

**Table 1:** Intake adjustments for several products within the same generic food item

Norkost 4 registered food product	Intake of Norkost 4 registered food product	Potential food products on the market	Additives	Adjusted intake
Beetroot salad	15 g	Beetroot salad brand A	E412, E415	5 g
		Beetroot salad brand B	E415, E466	5 g
		Beetroot salad brand C	E412, E415	5 g

To capture the most relevant exposure in the Norwegian diet, the 15 most used hydrocolloids on the Norwegian market, according to our previous study [30], were identified and mapped across all food products eaten by the Norkost 4 cohort. These included E401 Sodium alginate, E406 Agar, E407 Carrageenan, E410 Locust bean gum (LBG), E412 Guar gum, E413 Tragacanth, E414 Acacia gum, E415 Xanthan gum, E417 Tara gum, E418 Gellan gum, E440 Pectin, E460 Cellulose, E461 Methyl cellulose, E464 Hydroxypropylmethylcellulose (HPMC), and E466 CMC.

Food items were classified into 16 food groups, described in Table 2. Two food groups, eggs & egg products and spices, contained no products with hydrocolloids and were therefore excluded from the exposure analyses.

**Table 2:** Food groups used in the present analysis, with abbreviations used in figures and tables, and examples of products included in the food groups

Full name	Abbreviation	Examples
Beverages (including alcohol and excluding dairy products)	Beverages	Juice, soft drinks, alcoholic beverages, coffee, tea, milk analogues
Bread and crisp bread	Bread	White bread, wholegrain bread, crispbread, flatbread
Butter, margarine, oils, sauces and dressings	Fats & sauces	Butter, margarine, cooking oils, mayonnaise, dressings, gravies
Cakes, cookies and sweet baked goods	Cakes & cookies	Cakes, muffins, buns, cookies, waffles, pastries
Cereals and products thereof	Cereals	Oats, breakfast cereals, pasta, rice, flour, porridge
Cheeses	Cheeses	Hard cheese, soft cheese, cream cheese, blue cheese
Cream-based and cultured dairy products	Dairy (cream)	Yoghurt, sour cream, desserts
Fish and fish products	Fish prod.	Fresh fish, fish cakes, fish pudding, shellfish, canned fish
Fruit, berries and products thereof	Fruit prod.	Fresh fruit, berries, jam, dried fruit, fruit compote
Meat and meat products	Meat prod.	Fresh meat, processed meat, sausages, cold cuts
Milk and milk beverages	Milk prod.	Whole milk, skimmed milk, chocolate milk, iced coffee
Others	Others	Dietary supplements, vitamins, minerals, cooking ingredients
Snacks	Snacks	Crisps, popcorn, salted nuts, pastilles, chewing gum
Sugar, sweets and chocolate	Sugar & sweets	Sugar, chocolate, candy, syrup, sweeteners
Veg-friendly products	Veg-friendly	Plant-based meat and dairy alternatives (excluding beverages)
Vegetables, vegetable products, nuts, seeds and legumes	Vegetable prod.	Vegetables, processed vegetable-based products, potatoes, legumes, nuts, seeds

#### Concentrations of hydrocolloids

Concentrations of hydrocolloids in the food groups were obtained from EFSA’s re-evaluations of the additives included in this study, published between 2016 and 2018 [3–14]. To estimate realistic hydrocolloid intake, we used the mean concentrations reported in EFSA’s refined exposure assessments. These assessments estimate typical additive levels in foods based on industry-reported use levels and occurrence data, providing a more accurate basis for exposure calculations than maximum permitted levels. Accordingly, concentrations were only assigned to products where the specific hydrocolloid appeared in the ingredient list.

To assign food products a concentration of each additive when present, the following hierarchy was used: (1) EFSA food group values when available, (2) manually borrowed values from a closely related food group when no specific EFSA value existed, and (3) the median concentration for that additive across all products in our dataset when neither of the above applied. To evaluate the impact of uncertainty in concentration data, a sensitivity analysis was conducted for products assigned median concentrations, as this represented the most uncertain imputation step. Three concentration scenarios (Base, Lower, Upper) were defined based on whether imputed values used additive medians, Q25, or Q75; products with EFSA-based or borrowed concentrations remained unchanged across scenarios. Sensitivity was evaluated by examining the share of total exposure from imputed values, the proportion of each participant’s intake based on imputed concentrations, and how total exposure changed between scenarios using differences, ratios, and scatterplots. This sensitivity analysis is shown in the Supplementary information.

### Statistical analyses

All data processing, statistical analyses, and visualizations were performed in R (v. 4.5.3) and using the *tidyverse* (v. 2.0.0) package [40], with additional packages as cited below.

Hydrocolloid intake was estimated for each of the two recall days separately, resulting in two observations per participant per additive. Throughout this study, three distinct levels of exposure estimation are presented: (1) intake on consumption days (raw intake on days with positive consumption), (2) person-level intake (the two-day average per participant, presented as both population mean and consumer mean), and (3) long-term habitual intake, estimated using the R-package *Statistical Program to Assess Dietary Exposure* (SPADE) [41].

The distribution of intake on consumption days was visualized for each additive using stacked kernel density plots with a log-transformed x-axis. Food group contributions were shown as colored stacked curves, and the y-axis was scaled to reflect the absolute number of person-days rather than relative density.

Food group contributions to total hydrocolloid intake were further examined across demographic subgroups. For each additive, the percentage of total intake attributable to each food group was calculated separately by gender and age group (18–29, 30–44, 45–59, 60+). Food groups contributing less than 5% were excluded. Results were visualized as heatmaps using the package *pheatmap* (v.1.013) [42].

Associations between demographic characteristics (gender, age, BMI, and education) and consumption frequency were assessed using generalized estimating equations (GEE) with a binomial distribution, implemented in the *geepack* package (v. 1.3.13) [43] including gender, age, BMI, and education as covariates to account for the two correlated observations per participant. Results were reported as odds ratios (OR) with 95% confidence intervals. Associations with intake amount on consumption days were assessed at the person level using the Wilcoxon rank-sum test for gender, Spearman correlation for age and BMI, and the Kruskal–Wallis test for education. Statistical significance was set at *P* < 0.05.

To characterize high consumers, the top 5% of consumption days by intake level were identified for each additive separately. Demographic characteristics, proportion of women, mean age, mean BMI, and proportion with long higher education, were calculated for this group and compared with the overall population mean. Only additives with at least 30 observations in the top 5% were included in this analysis.

Co-occurrence between additives was assessed at the day level by calculating conditional probabilities, defined as the probability that additive B was consumed on a given day, given that additive A was consumed on the same day.

To estimate the habitual intake of the hydrocolloids, the R-package SPADE was used, developed by The National Institute for Public Health and the Environment in The Netherlands [41]. The 2-part model was fitted separately for men and women. Briefly, the model first estimates the probability of consumption as a function of age, then models the consumed amount. The probability of consumption is subsequently multiplied by the amount, and percentiles are obtained through Monte Carlo simulations (100 x n for each gender). The confidence intervals for each estimated level (median, mean, percentiles) were obtained using bootstrap sampling, according to the documentation, with 200 replications. Apart from age, no additional covariates were included in the modelling. To reliably estimate between-day intake variability within a person, SPADE needs at least 50 participants to have consumed the additive on both recall days; hence, we report SPADE output for only those seven additives where this was the case.

## Results

### Study population characteristics

A total of 1964 participants completed two 24-hour dietary recalls and were included in the study population. The mean BMI was 25.5 ± 4.3 kg/m^2^ and the mean age was 46.2 ± 16.3 years. Of the participants, 53% were women. Additional participant characteristics are presented in Table 3.

**Table 3:** Study population characteristics of the participants of the study

Variable	Level	*n* (%)
Gender	Female	1049 (53.4)
	Male	915 (46.6)
Education	< 14 years (Vocational/High School)	746 (38.0)
	1–4 years university/college: Short HE^a^	774 (39.4)
	Master/PhD: Long HE^a^	444 (22.6)
Age group (years)	< 30	375 (19.1)
	30–44	555 (28.3)
	45–59	557 (28.4)
	60+	477 (24.3)
BMI group (kg/m^2^)	< 18.5 (Underweight)	36 (1.8)
	18.5–24.9 (Normal weight)	959 (48.8)
	25.0–29.9 (Overweight)	646 (32.9)
	≥ 30 (Obese)	289 (14.7)
	Missing BMI information	34 (1.7)

^a^HE = Higher education.

Hydrocolloids were widely consumed across the study population. However, the frequency, amount, and food sources of exposure differed considerably between additives, shown in Table 4.

**Table 4:** Number of consumers, consumption day prevalence, raw intake metrics on consumption days and at person-level, and top three food sources for total and individual hydrocolloids. Total is the summary of intakes of the 15 hydrocolloids included in the study. Study population *n* = 1964

Additive			Intake on consumption days (mg/day)^a^			Intake on consumption days (mg/kg bw/day)		Person-level means (mg/day)^b^		
	Consumers, *n* (%)^c^	Consumption days (%)	Median	Mean	P95	Median	P95	Population mean	Consumer mean	Top 3 food sources
Total	1935 (99%)	92	313	496	1,596	4.1	20.6	459	466	Sugar & sweets (19%), Beverages (17%), Fruit prod. (15%)
E412 Guar gum	1810 (92%)	76	58	110	387	0.8	5.0	84	91	Fats & sauces (24%), Bread (19%), Beverages (15%)
E415 Xanthan gum	1445 (74%)	53	37	59	195	0.5	2.6	31	42	Fats & sauces (67%), Cakes & cookies (19%), Beverages (7%)
E440 Pectin	1185 (60%)	43	170	250	720	2.2	10.0	106	176	Fruit prod. (59%), Dairy (cream) (24%), Cakes & cookies (6%)
E407 Carrageenan	1166 (59%)	39	6	50	266	0.1	3.7	19	33	Dairy (cream) (40%), Cakes & cookies (25%), Fish prod. (12%)
E414 Gum arabic	988 (50%)	35	202	396	1,425	2.8	17.9	138	274	Sugar & sweets (56%), Beverages (37%), Cakes & cookies (6%)
E460 Cellulose	839 (43%)	27	5	29	102	0.1	1.4	8	19	Dairy (cream) (33%), Fats & sauces (32%), Bread (17%)
E410 LBG	772 (39%)	24	64	102	330	0.8	4.3	24	62	Dairy (cream) (36%), Meat prod. (15%), Cheeses (13%)
E401 Sodium alginate	707 (36%)	20	16	66	287	0.2	3.9	13	37	Fats & sauces (76%), Fish prod. (12%), Dairy (cream) (5%)
E466 CMC	556 (28%)	16	52	99	266	0.8	3.7	16	57	Beverages (38%), Dairy (cream) (23%), Cakes & cookies (18%)
E406 Agar	305 (16%)	9	2	15	24	0.0	0.2	1	8	Dairy (cream) (77%), Cakes & cookies (13%), Sugar & sweets (6%)
E418 Gellan gum	241 (12%)	7	45	61	168	0.6	2.5	4	36	Milk prod. (80%), Beverages (12%), Dairy (cream) (8%)
E464 HPMC	170 (9%)	5	84	159	600	1.2	8.4	7	83	Cakes & cookies (42%), Cheeses (29%), Bread (19%)
E413 Tragacanth	149 (8%)	4	8	41	146	0.1	1.7	2	21	Cakes & cookies (96%), Sugar & sweets (4%)
E417 Tara gum	73 (4%)	2	70	94	210	0.9	3.0	2	49	Dairy (cream) (86%), Cakes & cookies (13%), Veg-friendly (1%)
E461 Methyl cellulose	72 (4%)	2	66	126	378	0.8	6.1	2	63	Veg-friendly (73%), Cheeses (10%), Cakes & cookies (9%)

^a^Median, mean, and P95 calculated on consumption days only, at the day level. mg/kg bw estimated using reported body weight.

^b^Consumer mean: mean intake per person among those with ≥ 1 positive recall day, averaged across both days. Population mean: mean across all participants including non-consumers.

^c^Consumers defined as persons with at least one positive recall day. % consumption days calculated at day level (n obs = 2 × n persons).

99% of all participants had intake of at least one hydrocolloid over the two recall days, with hydrocolloid intake recorded on 92% of all individual recall days. On days when any hydrocolloid was consumed, the median total hydrocolloid intake was 313 mg/day (P95: 1,596 mg/day), corresponding to a median of 4.1 mg/kg bw/day (P95: 20.6 mg/kg bw/day). When averaged across both recall days per person (person level), the mean intake for the entire population was 459 mg/day, rising up to 466 mg/day when restricted to consumers only. The primary food group contributors to total exposure were sugar and sweets (19%), beverages (17%), and fruit and berry products (15%).

Patterns of intake varied widely across individual additives. Guar gum, xanthan gum, and pectin were the most frequently consumed additives, with 60–92% of participants reporting intake of these on at least one recall day. On consumption days, median intake was 58 mg/day for guar gum, 37 mg/day for xanthan gum, and 170 mg/day for pectin. Looking at the consumer level, the consumer mean intake was 91 mg/day for guar gum, 43 mg/day for xanthan gum, and 176 mg/day for pectin. The main food sources differed: guar gum intake was primarily derived from fats and sauces (24%) and bread (19%), xanthan gum almost exclusively from fats and sauces (67%), and pectin predominantly from fruit and berry products (59%) and cream-based dairy products (24%).

Carrageenan and gum arabic were consumed by approximately half of the participants (59% and 50%), but showed highly contrasting intake patterns. Carrageenan had a low median intake of 6 mg/day on consumption days, whereas gum arabic had a median of 202 mg/day with a P95 of 1,425 mg/day, and a two-day consumer mean of 274 mg/day, reflecting episodic but intense consumption primarily from sugar and sweets (56%) and beverages (37%). Cellulose, LBG, sodium alginate, and CMC showed moderate prevalence (28–43%), with median consumption-day intakes ranging from 5 to 64 mg/day, and two-day consumer means ranging from 19 to 62 mg/day.

Several additives were consumed by few participants (4–16%), including agar, gellan gum, HPMC, tragacanth, tara gum, and methyl cellulose. Despite low prevalence, some showed high intakes among those exposed; for instance, HPMC had a median consumption day intake of 84 mg/day, a P95 of 600 mg/day, and a two-day consumer mean of 83 mg/day, while tara gum and methyl cellulose had consumer means of 70 and 66 mg/day, respectively. Intake of tragacanth was almost exclusively attributed to cakes and cookies (96%), methyl cellulose predominantly from veg-friendly products (73%), and gellan gum primarily from milk products (80%).

### Food group sources shape intake distributions

**Fig. 1:**
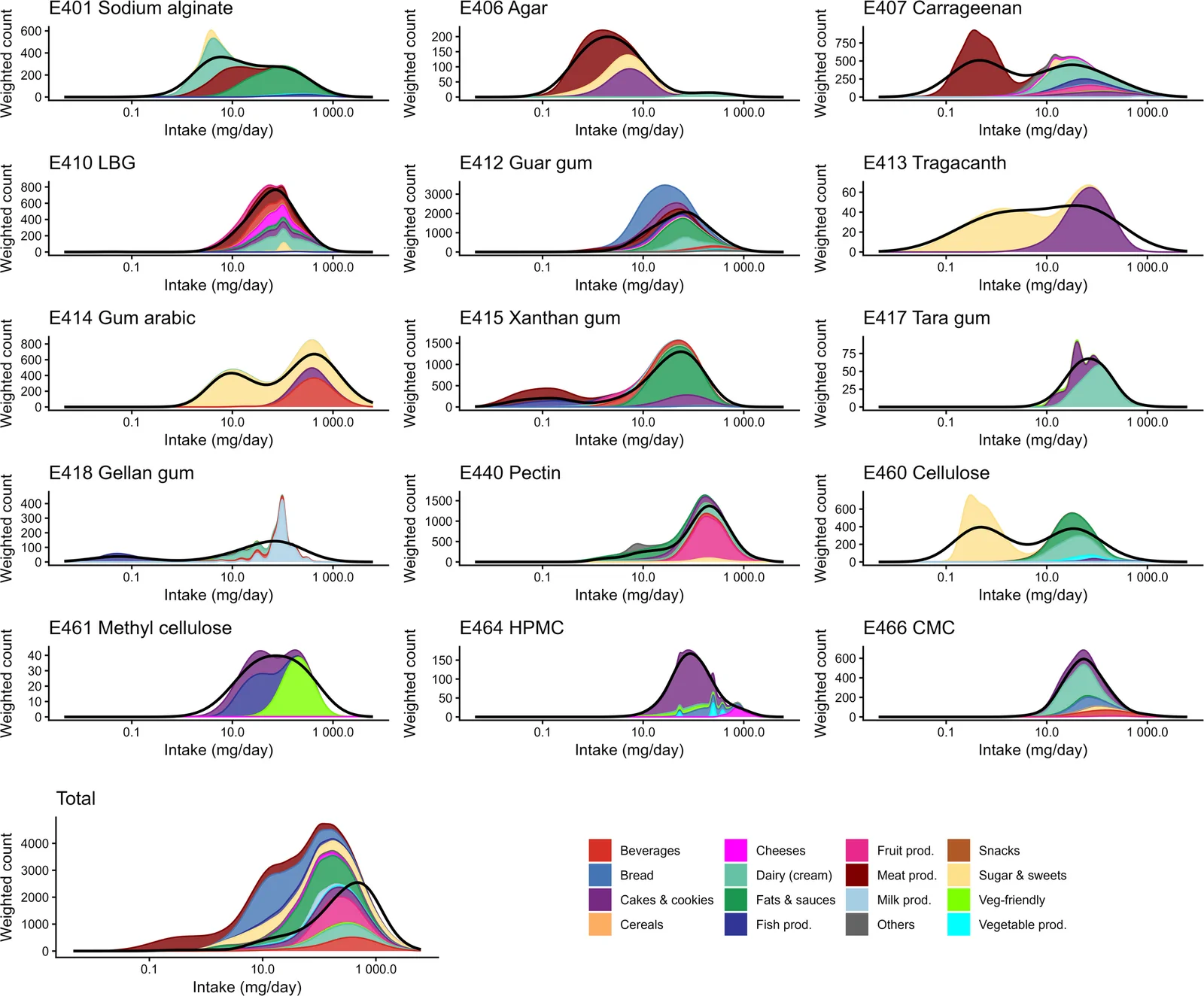
Intake distribution on consumption days for individual and total hydrocolloids, by food group. Colored curves show the contribution from each food group (stacked), and the black outline shows the total intake distribution from all food groups. Because a single person-day may contribute to multiple food group curves, the stacked curves may exceed the black outline

The distribution of hydrocolloid intake from different food groups on consumption days is shown in Fig. 1. For each additive, colored curves show the intake distribution from individual food groups, stacked to illustrate their relative contributions across the intake range, while the black outline shows the overall intake distribution. Because a single person-day can contribute to multiple food group curves simultaneously, the stacked curves may exceed the black outline at any given intake level.

Total hydrocolloid intake was broadly distributed and right-skewed, spanning several orders of magnitude from less than 1 mg/day to over 1,000 mg/day.

Some hydrocolloids showed unimodal distributions, suggesting relatively uniform intake patterns among consumers over the two days. This includes CMC, HPMC, LBG, guar gum, and pectin. Other additives showed pronounced bimodal distributions. This was especially true for carrageenan, gum arabic, and cellulose. For carrageenan, the bimodality depends on contributions from different food groups, where meat intake explains the lowest peak around 1 mg/day, while the other peak is explained by intake from several other food groups. The bimodality of gum arabic can also be explained by intake of different food groups, where the lower peak around 10 mg/day is dominated by sugar & sweets, and the other peak closer to 100 mg/day is explained by intake of beverages in addition to sugar & sweets. The bimodality seen for cellulose is also explained by different food groups, with sugar & sweets dominating the lower peak and dairy and fats & sauces dominating the other peak.

### Food group sources of exposure differ by age

**Fig. 2:**
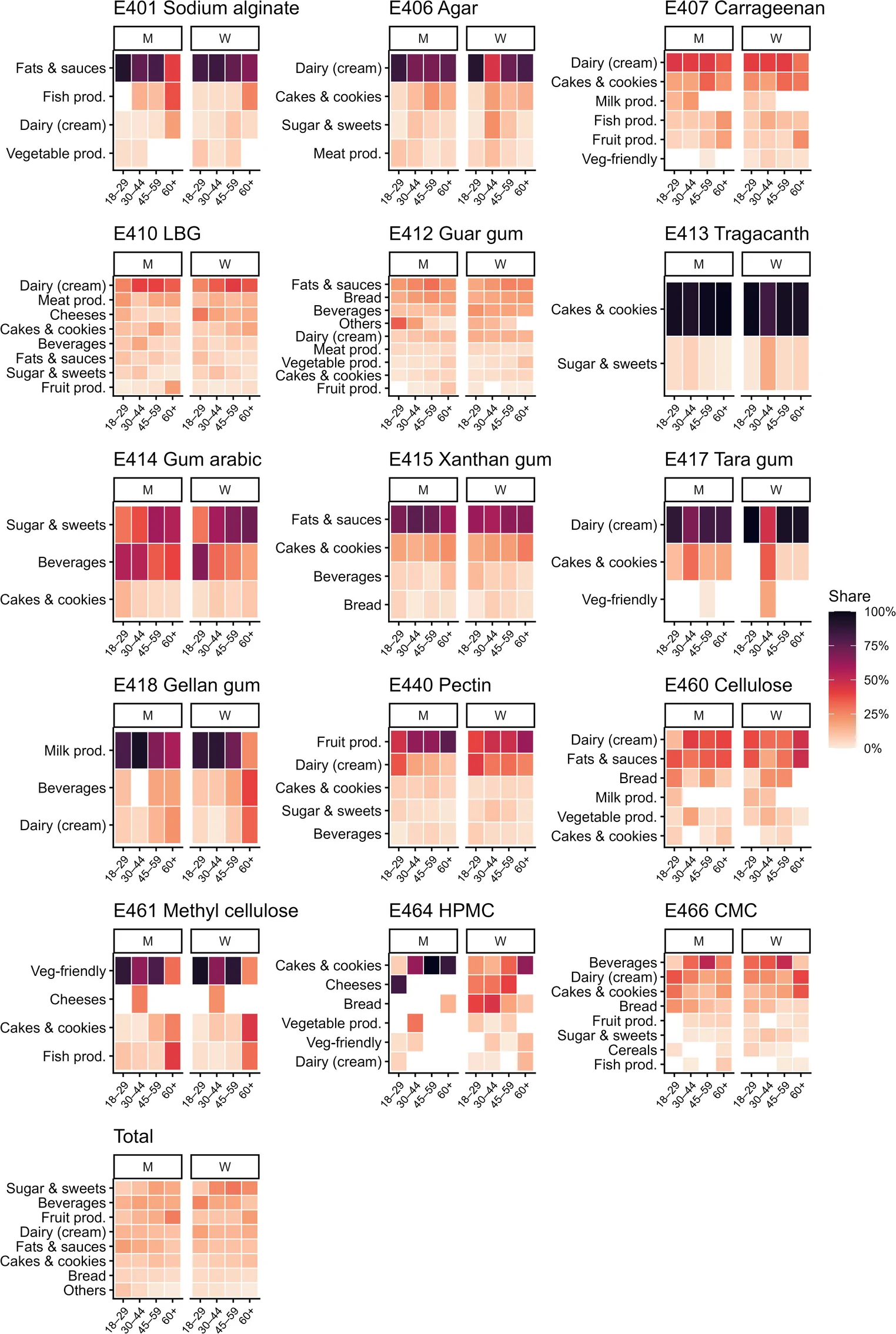
Food group contributions to total hydrocolloid intake by gender and age group. Cell color represents the percentage of total intake (mg/day) attributed to each food group within each gender and age stratum, calculated strictly for positive consumption days. Only food groups contributing ≥ 5% in at least one stratum are shown

Food group contributions to total hydrocolloid intake, stratified by gender and age group, are shown in Fig. 2. For most additives, food group contributions were broadly stable across gender and age groups. However, some age-related shifts were apparent for individual additives.

For gum arabic, beverages were the dominant source among younger age groups, while sugar and sweets became increasingly dominant with age. For pectin, the contribution from fruit and berry products increased with age. For gellan gum, milk products were the dominant sources, particularly among younger age groups. For cellulose, veg-friendly products contributed more among younger participants, while fish products and cakes & cookies became more prominent among older age groups. For total hydrocolloid exposure, no clear age- or gender-related differences in food group contributions were apparent.

### Demographics and hydrocolloid exposure

**Fig. 3:**
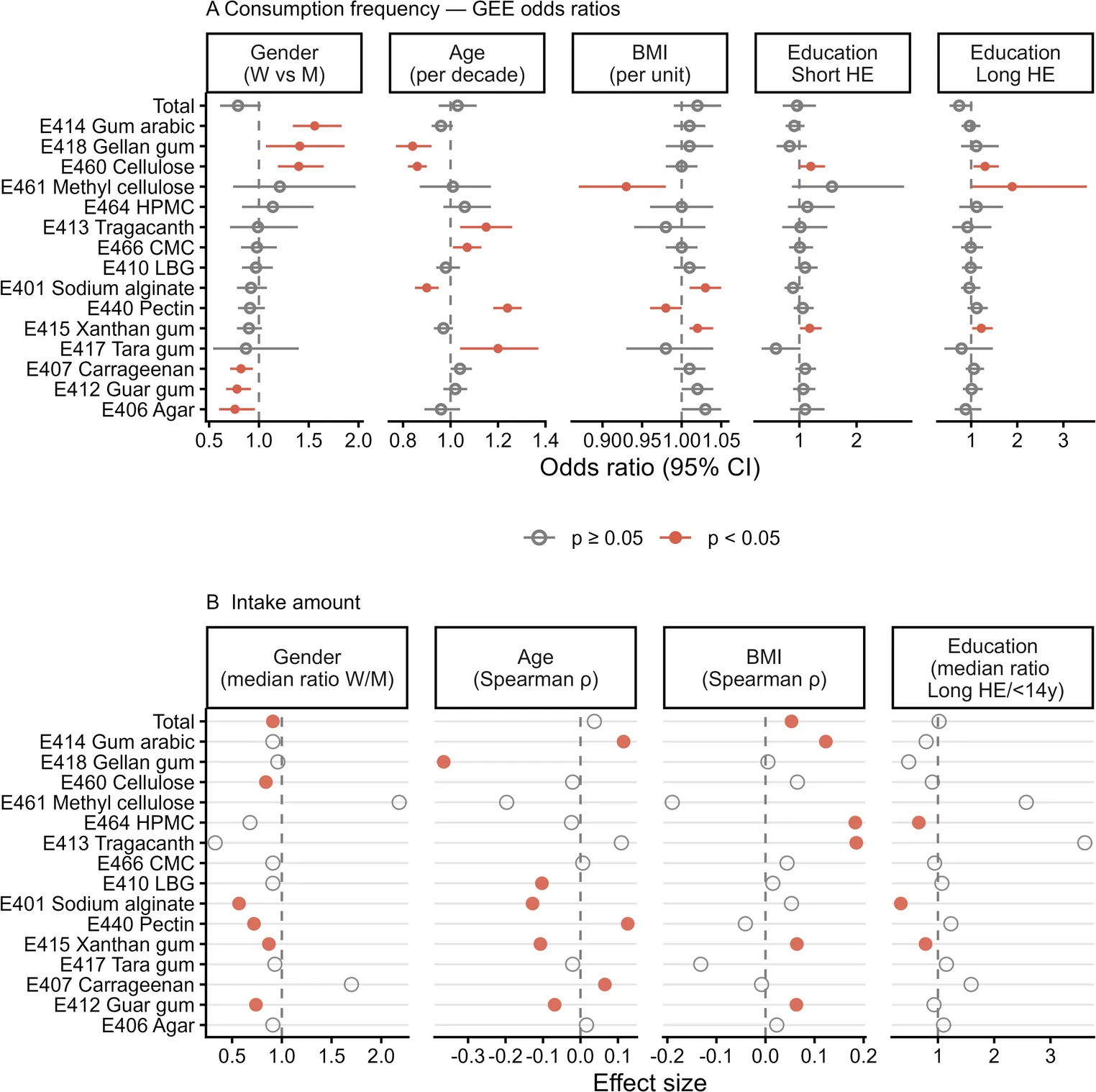
Associations between demographic characteristics and hydrocolloid consumption. **A**: Odds ratios (95% CI) for the probability of consuming a hydrocolloid on a given recall day, estimated using generalized estimating equations (GEE) with an exchangeable correlation structure to account for the two repeated measures per person. Contrasts shown: women vs. men, per 10-year increase in age, per unit increase in BMI, and short or long higher education (HE) vs. high school or less (≤ 14 years) as reference. **B**: Effect sizes for associations between demographic characteristics and intake amount among consumers (persons with ≥ 1 positive recall day), assessed using Wilcoxon rank-sum test (gender: median ratio women/men), Spearman rank correlation (age and BMI: ρ), and Kruskal-Wallis test (education: median ratio long higher education vs. high school). Note that panel A reflects consumption probability across both recall days, whereas panel B reflects intake amount among consumers only, averaged over the two recall days. Red filled circles indicate statistically significant associations (*p* < 0.05)

To examine whether hydrocolloid exposure varied across demographic subgroups, we assessed associations between gender, age, BMI, and education and both consumption frequency and intake amount, shown in Fig. 3. Consumption probability was modelled using GEE with a binomial distribution, accounting for the two correlated recall days per participant, and results are presented as odds ratios (Panel A). Associations with intake amount were assessed at the person level using non-parametric tests, and results are presented as median ratios (gender), Spearman correlation coefficients (age and BMI) and median ratios between education groups (Panel B).

Women had significantly higher odds of consuming gum arabic, gellan gum and cellulose, while odds were lower for carrageenan, guar gum and agar. Age was positively associated with increased consumption probability of pectin, tara gum, tragacanth and CMC, and negatively associated with gellan gum, cellulose and sodium alginate. Higher BMI was associated with increased consumption probability of sodium alginate and xanthan gum, and decreased probability of methyl cellulose. Both short and long higher education were associated with higher odds of consuming cellulose and xanthan gum, while long higher education was associated with higher odds of consuming methyl cellulose.

Total hydrocolloid intake was significantly lower in women than in men, and increased with BMI. Among individual additives, women had significantly lower intakes of cellulose, pectin, xanthan gum, guar gum, and sodium alginate. Age was positively associated with intakes of gum arabic, pectin, and carrageenan, and negatively associated with gellan gum, LBG, sodium alginate, xanthan, gum and guar gum. BMI was positively associated with intake of gum arabic, tragacanth, HPMC, xanthan gum, and guar gum. Participants with long higher education (HE) had significantly lower intake of HPMC, sodium alginate, and xanthan gum. The exact association statistics are presented in Supplementary information Table 7.

### Characteristics of high hydrocolloid consumers

Demographic characteristics of individuals with the highest observed intake on at least one recall day (top 5% of consumption days per additive) are shown in Table 5. These findings reflect acute high exposure. Compared to the population mean (53% women, mean age 46 years, mean BMI 26, 23% with long HE), the demographic profile of high-exposure individuals varied across additives.

High consumers of gum arabic and carrageenan were more often women (60% and 55%, respectively) and older than the cohort mean, while high consumers of sodium alginate, LBG, guar gum and CMC had a lower proportion of women (33–38% women) and younger mean age. High consumers of cellulose were also predominantly women (57%) but younger than the cohort mean (mean age 39 years).

BMI was slightly elevated among high consumers of sodium alginate, gum arabic and xanthan gum (all 27) compared to the cohort mean (26). Regarding education, high consumers of carrageenan and cellulose had a higher proportion with long HE (28% and 27%) than the cohort mean, while high consumers of sodium alginate, LBG, and CMC had notably lower proportions (13%, 13% and 14%).

**Table 5:** Demographic characteristics of individuals with the highest observed single-day intake (top 5% of consumption days) for total and individual hydrocolloids, compared to the study population mean. Only additives with at least 30 observations in the top 5% are included

Additive	Women (%)	Mean age (years)	Mean BMI	Long HE (%)
Cohort mean	53	46	26	23
Total	48	43	26	20
E401 Sodium alginate	33	44	27	13
E407 Carrageenan	55	47	25	28
E410 LBG	33	45	26	13
E412 Guar gum	37	43	26	20
E414 Gum arabic	60	48	27	14
E415 Xanthan gum	40	43	27	18
E440 Pectin	40	46	25	25
E460 Cellulose	57	39	25	2
E466 CMC	38	45	26	14

### Co-consumption of multiple hydrocolloids is common

**Fig. 4:**
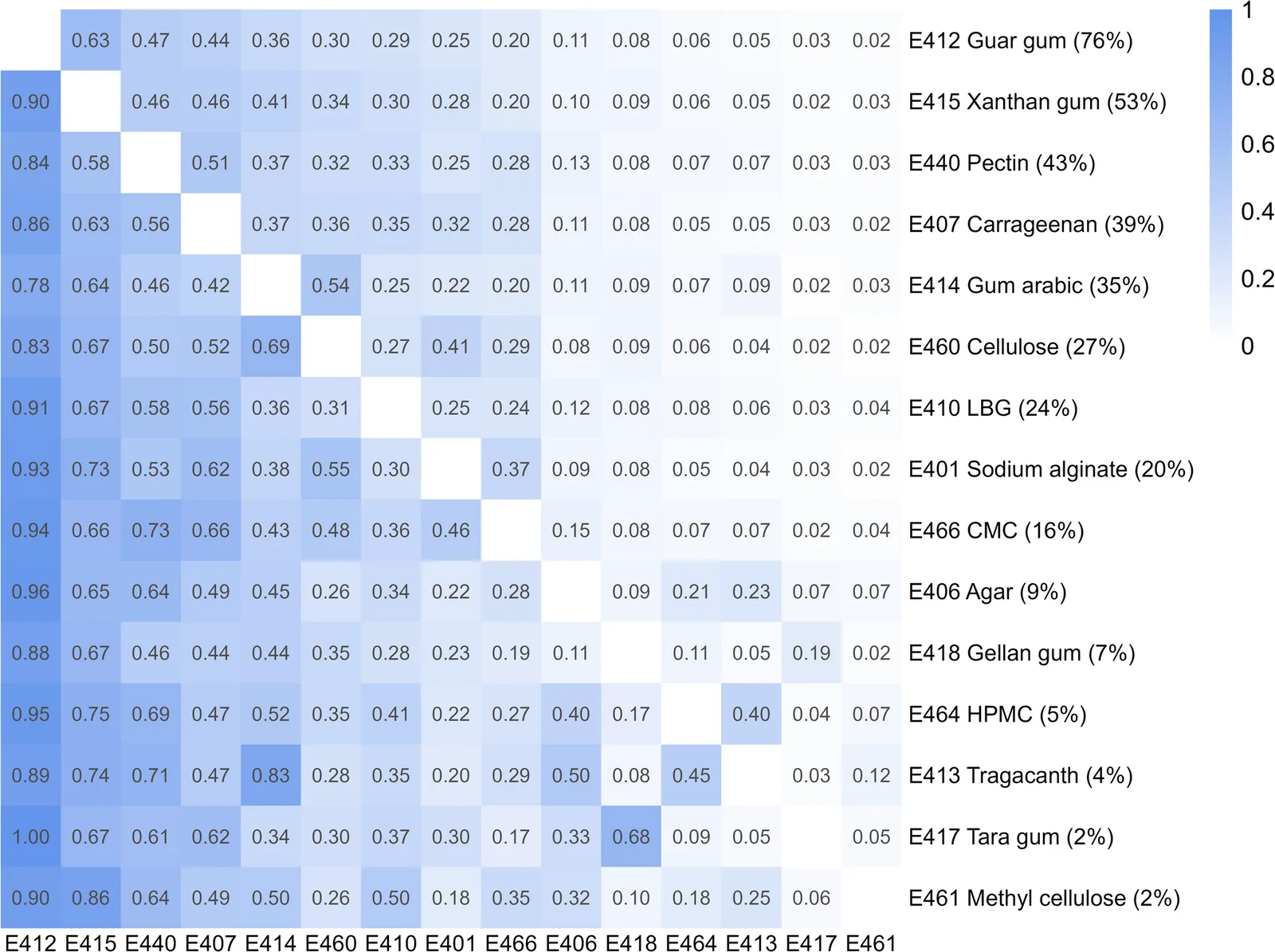
Co-consumption matrix for hydrocolloid additives. Each cell represents the conditional probability of consuming the column additive on a given day, given that the row additive was consumed on that same day, P(column | row), calculated at the recall-day level. Additives are ordered by consumption prevalence (highest to lowest), indicated by the percentage of person-days with positive intake shown in parentheses

To evaluate co-consumption of additives on the same day, the conditional probability of being exposed to two additives was assessed, as shown in Fig. 4. Values represent the probability that additive B was consumed given that additive A was consumed, with rows sorted by prevalence.

Overall, co-occurrence was high among the most prevalent additives, largely reflecting their high individual prevalence. Given the consumption of guar gum (76% prevalence), the probability of also consuming xanthan gum was 0.63, pectin 0.47, and carrageenan 0.44. Conversely, given consumption of any of the less prevalent additives, the probability of also consuming guar gum was very high, exceeding 0.78 for all additives, consistent with its high population prevalence.

Several additive pairs showed notably elevated conditional probabilities without being regularly consumed individually. Tara gum and gellan gum showed a conditional probability of 0.68; given consumption of tara gum, there was a 68% probability of also consuming gellan gum on the same day. A similar pattern was seen for tragacanth, where the probability of also consuming gum arabic when tragacanth was consumed was 0.83, even though intake of these additives only occurred on 14 and 4% of all recall days, respectively.

### Habitual intake

Seven additives met SPADE’s criterion of having ≥ 50 both-day consumers and were thus included in the analysis, and the whole study population was included. Consistent with the raw exposure estimates, habitual intake varied considerably between additives, as presented in Table 6. For men, the highest median habitual intake was observed for pectin (84 mg/day), closely followed by guar gum (82 mg/day). For women, the highest median habitual intake was observed for gum arabic (80 mg/day), followed by pectin (74 mg/day). The highest 95th percentile intake was observed for gum arabic in both genders, at 554 mg/day for men and 658 mg/day for women.

**Table 6:** Habitual intake of hydrocolloids estimated with SPADE (see text)

Additive	Men			Women		
	Median mg/day (95% CI)	Mean mg/day (95% CI)	P95 mg/day (95% CI)	Median mg/day (95% CI)	Mean mg/day (95% CI)	P95 mg/day (95% CI)
E407 Carrageenan	17 (8–28)	37 (26–49)	130 (82–178)	18 (9–28)	41 (28–57)	152 (92–217)
E410 LBG	16 (13–20)	28 (24–32)	92 (74–109)	16 (13–19)	21 (19–24)	61 (50–70)
E412 Guar gum	83 (76–90)	99 (91–108)	223 (190–260)	61 (56–67)	71 (65–78)	154 (124–182)
E414 Gum arabic	55 (42–69)	140 (117–163)	554 (440–667)	80 (61–99)	175 (153–196)	658 (549–766)
E415 Xanthan gum	30 (26–33)	37 (34–41)	95 (81–107)	25 (22–28)	28 (25–30)	58 (49–69)
E440 Pectin	84 (72–98)	120 (108–133)	358 (315–396)	74 (62–86)	95 (84–105)	250 (206–288)
E460 Cellulose	5 (2–8)	12 (8–17)	47 (27–67)	5 (2–8)	15 (11–19)	62 (43–76)

Confidence intervals (CI) were estimated by bootstrap sampling. *n* = 1049 for women, *n* = 915 for men.

## Discussion

Our comprehensive mapping of hydrocolloid intake in Norway found that hydrocolloid exposure is common, with 99% of participants consuming at least one of the 15 most used hydrocolloids during the two recall days. However, both the frequency and intake amounts varied greatly between individual hydrocolloids, largely driven by their specific food sources. Furthermore, co-consumption of multiple hydrocolloids on the same day was highly prevalent, particularly for the most frequently encountered additives. This co-consumption aligns well with previous market mapping in Norway [30]. Here we showed that 60% of foods containing an additive within the class of Emulsifiers, thickeners and stabilizers, contained more than one, frequently appearing in specific combinations.

Additives showed clear but disparate demographic consumer profiles in the present study. Nevertheless, the associations between demographics, consumption probability, and intake volume were highly additive-specific. Most notably, the characteristics of the highest consumers differed substantially across the various hydrocolloids. This lack of consistency strongly indicates that there is no single, homogeneous profile of an “extreme consumer” of hydrocolloids in the population.

To our knowledge, this is the first mapping of hydrocolloid intake in a nationally representative cohort. However, exposure to food additives in general, including some of the hydrocolloids, has been mapped in the NutriNet-Santé cohort in France by Chazelas and colleagues in 2021 [37] and in EFSA re-evaluations [3–14].

Overall hydrocolloid exposure levels quantified in the present study were substantially lower than the refined exposure assessments constructed by EFSA [3–14]. For instance, the median intake of guar gum on positive consumption days in our cohort was 0.8 mg/kg bw/day, which is below EFSA’s refined non-brand-loyal exposure assessment span of 7–273 mg/kg bw/day [6]. Even high consumers at the 95th percentile (5 mg/kg bw/day on consumption days) in our cohort remain below these estimates. This trend of lower exposure was highly consistent across all analyzed hydrocolloids. This is most likely due to methodological differences, as discussed below.

Notably, carrageenan is currently the only hydrocolloid in this assessment with an established ADI, namely 75 mg/kg bw/day [12]. The mean intake among Norwegian consumers on consumption days was a mere 0.1 mg/kg bw/day, with the 95th percentile reaching 3.7 mg/kg bw/day. This equals a mean of 50 mg/day among consumers at the person-level. In comparison, EFSA’s refined estimates for carrageenan span from 10 to 17 mg/kg bw/day. Estimates from SPADE were even lower (mean intake of 37–41 mg/day), underscoring that regular intake in the Norwegian population is safely below the regulatory safety thresholds.

Compared with the French NutriNet-Santé cohort [37], which utilized a comparable dietary survey approach, the Norwegian cohort showed notable differences in exposure. A clear diverging pattern emerged between exposure prevalence and actual intake volumes. For instance, guar gum exposure was substantially more widespread in Norway (92% vs. 68% in France), reflecting its ubiquity in Norwegian foods, yet the actual population mean intake was much lower (84 mg/day vs. 310 mg/day). A similar trend of high prevalence but modest intake volumes were observed for xanthan gum.

Conversely, carrageenan and pectin exhibited a highly episodic exposure pattern in the Norwegian cohort. Fewer Norwegians were exposed to carrageenan on any given day compared to the French (59% vs. 78%), and the modeled habitual mean intake remained similar (37–41 mg/day vs. 46 mg/day). This episodic peak was even more pronounced for pectin; despite a lower daily prevalence (60% versus 79%), intake on consumption days reached a mean of 250 mg/day, peaking at a 95th percentile of 720 mg/day. The population mean in our cohort was 106 mg/day, and 200 mg/day in the French cohort. Together, these findings demonstrate that while habitual baseline exposure over time appears lower in Norway than in France, Norwegian consumers face highly concentrated exposures on the specific days these additives are actually ingested.

While it is expected that cross-national comparisons might reflect genuine differences in exposure patterns between countries, it should be noted that cross-study comparisons of additive intake are challenged by differences in dietary data collection, additive-content mapping, episodic consumption, statistical modelling, and additive-specific food-group and consumer patterns.

In a comprehensive review of methodologies used to estimate food additive intake such challenges were addressed [44]. In this review they outlined a multi-tiered framework for exposure mapping: Tier 1 utilizes theoretical food consumption combined with maximum permitted levels (MPL) of additives, representing a strict “worst-case” scenario; Tier 2 pairs actual food consumption data with MPLs; whereas Tier 3 combines actual consumption with actual additive usage levels or quantitative chemical analyses. Additionally, they categorized these assessments into different methodological approaches, namely per capita methods, total diet studies, and individual dietary surveys. Because the various studies used for comparison with our work represent fundamentally different tiers and methodologies, direct comparisons between them should be interpreted with caution.

For example, our results are comparable to the lower-bound estimates of CMC in the US cohorts analyzed by Shah and colleagues in 2017 [38]. However, their methodology highlights how drastically the choice of concentration data and framework affects the results; when assuming a “worst-case” scenario where all foods contain maximum permitted use levels (MUL), they estimated a mean intake of 24–30 mg/kg bw/day. In contrast, when they applied a market volume disappearance method, the per capita exposure dropped to 2 mg/kg bw/day. This latter market-based estimate aligns far better with our refined results from the Norwegian cohort (median intake of 0.8 mg/kg bw/day and a 95th percentile of 3.7 mg/kg bw/day), underscoring that deterministic simulations based on maximum allowances tend to heavily overestimate actual intake.

A systematic review by Hall and Trakman from 2025 [45] of estimations of food additive intakes also highlights the challenges and study differences of such research. They emphasize that since no gold-standard methodology exists, providing quantitative additive data on food labels is key to improving human exposure research and risk assessments. This need for quantitative data is particularly critical for hydrocolloids, where laboratory-based chemical quantification in complex food matrices remains uniquely challenging due to their complex polysaccharide structures.

While this present study possesses several methodological advantages, such as employing an open-ended dietary survey and isolating additive intakes to specific products, certain limitations must be addressed.

The primary challenge, in addition to the lack of accurate concentration data, stems from the inherent nature of high-detail dietary surveys: participants do not always recall or know the exact brand or specific product consumed. Because standard or “typical” baseline levels for additives within general food categories do not exist, we addressed these gaps by distributing the estimated intake across all relevant matching products. Narratively, this approach has a dual effect on our findings. First, it likely artificially broadens the estimated range of additive exposure, making it appear as though participants encounter a wider variety of additives than they do in reality. Second, it simultaneously dilutes the calculated volume of the specific additive actually consumed, meaning the real intake magnitude for certain individuals might be underestimated. Furthermore, general recall bias remains an inherent risk common to all such self-reported methods.

This study introduces a novel approach by modeling habitual intake for food additives. Because our framework relies on short-term recalls rather than long-term frequency data, we were unable to statistically identify true “never-consumers”, despite our assumption that such individuals exist in the population. Furthermore, the statistical architecture of the two-part SPADE model does not account for a potential correlation between consumption frequency and the portion amounts consumed on consumption days. To balance this complexity and account for the multi-layered uncertainties in the raw data, we deliberately chose a simplified modeling framework. We excluded potential low-impact covariates and left the data unweighted to national averages, prioritizing a transparent and reliable baseline model over an overly complex statistical modelling. Lastly, validating our findings with traditional lifetime-exposure methods, such as food frequency questionnaires (FFQs), is challenging, as such methods cannot capture the product-specific details necessary for additive mapping.

In conclusion, while hydrocolloid exposure is widespread among Norwegian adults, individual intake patterns vary significantly, and we find no evidence of a homogenous group of “acute extreme consumers”. Given that our analysis is based on a nationally representative dietary survey, these findings carry high generalizability to the broader Norwegian adult population and provide a realistic baseline for Nordic dietary patterns. Crucially, our estimated intakes are well below EFSA’s estimates and the ADI for carrageenan. However, as nutritional science shifts toward exploring non-traditional endpoints like the gut microbiota, the methods used to capture additive exposure must adapt. The current lack of publicly available, brand-specific concentration data remains a major bottleneck. By contrasting our refined findings against conservative regulatory simulations, this study demonstrates that actual intake quantities are lower than previously assumed, highlighting the critical need for transparent, quantitative label data to enable accurate, clinically relevant risk assessments.

Supplementary information.

## Supplementary Information

Below is the link to the electronic supplementary material.

Supplementary Material 1

## Data Availability

The dietary data from Norkost 4 were obtained from the University of Oslo / Norwegian Directorate of Health; restrictions apply to the availability of these data, which were used under license for the current study and are not publicly available. Product information, ingredient lists, and concentration data were derived from the European Food Safety Authority (EFSA) journal, the VetDuAt.no product database (Tradesolutions), and the Kassalapp database. These third-party databases are available through their respective public platforms or commercial agreements.

## Declarations

### The use of artificial intelligence

Artificial intelligence (AI) was used for coding assistance and text refinement. The authors of the study take full responsibility for its content, claims, and references.
