# Supplementary material for: Hydrocolloid food additive intake in the Norwegian population: a dietary exposure assessment
Source: Eur J Nutr. 2026 Nov 11;65(8):302. doi: 10.1007/s00394-026-04159-5 (PMC13755042; doi:10.1007/s00394-026-04159-5)
Supplement: Supplementary file 1 [file 394_2026_4159_MOESM1_ESM.docx]

# Supplementary information

Sensitivity analysis of concentration imputation
A sensitivity analysis was conducted to evaluate how imputed hydrocolloid concentrations influenced the exposure (average mg/day) estimates. In the base scenario, only 0.8% of total exposure came from products where concentrations were imputed using additive level medians, indicating that the exposure assessment was almost entirely driven by EFSA based or manually borrowed values.

At the individual level, the proportion of exposure originating from imputed concentrations was generally very small. The median was 0.0%, and the 90th and 95th percentiles were < 5.0% (0.9% and 4.6%, respectively). Only a small number of individuals showed higher proportions (99th percentile: 16.9%), reflecting occasional reliance on imputed concentrations for specific combinations of additives and products.

When varying the imputed values between the additive‑specific 25th and 75th percentiles (Lower and Upper scenarios), total exposure remained stable. For 95% of participants, the Upper scenario increased total exposure by no more than 4% compared with the Base scenario (P95 upper/base = 1.04). The Lower scenario produced nearly identical values for 95% of individuals (P95 lower/base = 1.00). Scatterplots of Base vs Upper and Base vs Lower exposure showed points closely aligned with the diagonal, shown in Figure 5, confirming that the results were robust to plausible variation in imputed concentrations.

To identify which additives contributed most to scenario differences, we examined additive‑specific exposure ratios. The largest deviations were observed for agar (E406), which had the greatest variation in reported concentrations. Even in these cases, the changes affected only a very small number of individuals and did not meaningfully influence population‑level exposure estimates.

**Fig. 5:** Sensitivity of total hydrocolloid exposure to concentration‑imputation assumptions. Each point represents one individual’s total hydrocolloid exposure (mg/day). Base exposure uses EFSA food‑group concentrations when available, borrowed values when needed, and additive medians only when no analogous EFSA value existed. The lower and upper scenarios replace these median‑imputed concentrations with the 25th or 75th percentile of observed EFSA/borrowed values for the same additive. Close alignment with the identity line indicates minimal impact on total exposure.

## Table 7 Consumption probability and intake amounts between demographic groups

|  | **Consumption probability**  **y — OR (95% CI), p*^1^*** | | | | | **Intake amount among consumers — effect size, p*^2^*** | | | |
| --- | --- | --- | --- | --- | --- | --- | --- | --- | --- |
| Additive | Gender (W vs M) | Age (per decade) | BMI (per unit) | Short HE vs. ≤14y | Long HE vs. ≤14y | Gender (ratio W/M) | Age (ρ) | BMI (ρ) | Education (ratio Long HE/≤14y) |
| E401 Sodium alginate | 0.92 (0.78–1.08), 0.307 | 0.9 (0.85–0.95), <0.001 | 1.03 (1.01–1.05), 0.001 | 0.89 (0.74–1.07), 0.205 | 0.96 (0.78–1.20), 0.738 | 0.57, <0.001 | -0.128, <0.001 | 0.053, 0.166 | 0.34, <0.001 |
| E406 Agar | 0.76 (0.60–0.96), 0.022 | 0.96 (0.89–1.04), 0.366 | 1.03 (1.00–1.05), 0.063 | 1.1 (0.84–1.44), 0.476 | 0.88 (0.64–1.22), 0.451 | 0.91, 0.077 | 0.016, 0.780 | 0.023, 0.691 | 1.1, 0.850 |
| E407 Carrageenan | 0.82 (0.71–0.94), 0.005 | 1.04 (1.00–1.09), 0.073 | 1.01 (0.99–1.03), 0.227 | 1.1 (0.93–1.29), 0.259 | 1.06 (0.88–1.28), 0.534 | 1.7, 0.068 | 0.065, 0.027 | -0.008, 0.797 | 1.59, 0.367 |
| E410 LBG | 0.97 (0.83–1.14), 0.708 | 0.98 (0.94–1.04), 0.549 | 1.01 (0.99–1.03), 0.398 | 1.1 (0.92–1.32), 0.285 | 0.99 (0.80–1.24), 0.940 | 0.91, 0.091 | -0.103, 0.004 | 0.015, 0.678 | 1.07, 0.646 |
| E412 Guar gum | 0.78 (0.67–0.92), 0.003 | 1.02 (0.97–1.07), 0.351 | 1.02 (1.00–1.04), 0.050 | 1.07 (0.89–1.28), 0.458 | 1.01 (0.82–1.25), 0.920 | 0.74, <0.001 | -0.069, 0.003 | 0.063, 0.008 | 0.93, 0.113 |
| E413 Tragacanth | 0.99 (0.71–1.39), 0.976 | 1.15 (1.04–1.26), 0.004 | 0.98 (0.94–1.03), 0.424 | 1.02 (0.70–1.49), 0.923 | 0.92 (0.59–1.44), 0.718 | 0.33, 0.070 | 0.109, 0.185 | 0.185, 0.024 | 3.61, 0.555 |
| E414 Gum arabic | 1.56 (1.34–1.83), <0.001 | 0.96 (0.92–1.01), 0.109 | 1.01 (0.99–1.03), 0.256 | 0.91 (0.76–1.09), 0.298 | 0.97 (0.79–1.20), 0.806 | 0.91, 0.645 | 0.115, <0.001 | 0.123, <0.001 | 0.79, 0.110 |
| E415 Xanthan gum | 0.9 (0.78–1.03), 0.125 | 0.97 (0.93–1.01), 0.143 | 1.02 (1.01–1.04), 0.007 | 1.18 (1.01–1.39), 0.039 | 1.22 (1.02–1.47), 0.034 | 0.87, 0.006 | -0.107, <0.001 | 0.064, 0.016 | 0.78, 0.040 |
| E417 Tara gum | 0.87 (0.54–1.40), 0.574 | 1.2 (1.04–1.37), 0.010 | 0.98 (0.93–1.04), 0.598 | 0.59 (0.34–1.02), 0.060 | 0.79 (0.42–1.47), 0.460 | 0.93, 0.842 | -0.021, 0.860 | -0.132, 0.268 | 1.15, 0.865 |
| E418 Gellan gum | 1.41 (1.07–1.86), 0.016 | 0.84 (0.77–0.92), <0.001 | 1.01 (0.98–1.04), 0.532 | 0.83 (0.60–1.13), 0.226 | 1.11 (0.78–1.60), 0.561 | 0.96, 0.807 | -0.365, <0.001 | 0.005, 0.936 | 0.48, 0.263 |
| E440 Pectin | 0.91 (0.79–1.06), 0.225 | 1.24 (1.18–1.30), <0.001 | 0.98 (0.96–1.00), 0.031 | 1.06 (0.90–1.25), 0.477 | 1.12 (0.92–1.36), 0.265 | 0.72, <0.001 | 0.126, <0.001 | -0.041, 0.163 | 1.23, 0.227 |
| E460 Cellulose | 1.4 (1.19–1.65), <0.001 | 0.86 (0.82–0.90), <0.001 | 1 (0.98–1.02), 0.915 | 1.2 (1.00–1.45), 0.047 | 1.3 (1.05–1.60), 0.017 | 0.84, 0.011 | -0.021, 0.545 | 0.065, 0.063 | 0.9, 0.332 |
| E461 Methyl cellulose | 1.21 (0.74–1.97), 0.444 | 1.01 (0.87–1.17), 0.869 | 0.93 (0.87–0.98), 0.013 | 1.57 (0.87–2.83), 0.131 | 1.89 (1.01–3.51), 0.045 | 2.18, 0.535 | -0.197, 0.097 | -0.19, 0.112 | 2.57, 0.216 |
| E464 HPMC | 1.14 (0.83–1.55), 0.415 | 1.06 (0.97–1.17), 0.189 | 1 (0.96–1.04), 0.938 | 1.14 (0.80–1.62), 0.477 | 1.12 (0.74–1.69), 0.603 | 0.68, 0.097 | -0.024, 0.755 | 0.183, 0.017 | 0.66, 0.018 |
| E466 CMC | 0.98 (0.82–1.18), 0.836 | 1.07 (1.01–1.13), 0.023 | 1 (0.98–1.02), 0.924 | 1.01 (0.82–1.24), 0.944 | 0.99 (0.78–1.26), 0.944 | 0.91, 0.139 | 0.006, 0.893 | 0.044, 0.305 | 0.94, 0.295 |
| *^1^*OR: odds ratio from GEE model with exchangeable correlation structure. Reference category for education: compulsory education (≤14 years). | | | | | | | | | |
| *^2^*Gender: median ratio (women/men) from Wilcoxon rank-sum test. Age and BMI: Spearman ρ. Education: median ratio (long higher education/compulsory education) from Kruskal-Wallis test. | | | | | | | | | |
